# Editorial: Monitoring Pathophysiology in the Injured Brain

**DOI:** 10.3389/fneur.2018.00193

**Published:** 2018-03-26

**Authors:** Eric Peter Thelin, Adel Helmy, David W. Nelson, Niklas Marklund

**Affiliations:** ^1^Division of Neurosurgery, Department of Clinical Neurosciences, University of Cambridge, Cambridge Biomedical Campus, Cambridge, United Kingdom; ^2^Department of Clinical Neuroscience, Karolinska Institute, Stockholm, Sweden; ^3^Department of Physiology and Pharmacology, Section of Perioperative Medicine and Intensive Care, Karolinska Institute, Stockholm, Sweden; ^4^Skane University Hospital, Department of Clinical Sciences Lund, Neurosurgery, Lund University, Lund, Sweden; ^5^Department of Neuroscience, Neurosurgery, Uppsala University, Uppsala, Sweden

**Keywords:** traumatic brain injury, subarachnoid hemorrhage, monitoring, biomarkers, neurocritical care

**Editorial on the Research Topic**

**Monitoring Pathophysiology in the Injured Brain**

## Introduction

Brain injuries can be caused by, for instance, spontaneous hemorrhages, thromboembolic incidents or traumatic events, and are considerable sources of morbidity and mortality (1). Brain injuries commonly result in immense socioeconomic consequences due to the acute as well as persisting neurological deficits (2). To date, there are currently few pharmacological therapies of proven clinical benefit targeting the underlying pathophysiology occurring in the aftermath of subarachnoid hemorrhage (SAH) (3), stroke (4), and traumatic brain injury (TBI) (5). Although these brain injuries are exceedingly heterogeneous, some common pathophysiological phases may be identified. At disease onset, the primary ictus may cause initial neuronal, glial, and vascular injury, which is then followed by complex pathophysiological responses. The initial tissue injury is then exacerbated by secondary insults, occurring in the vulnerable brain during the first post-injury period (6). The secondary injury cascades include among others neuroinflammation, energy failure, hypoxia, and inadequate cerebral perfusion (7). By monitoring multiple intracerebral as well as systemic parameters across a range of modalities, the time course of these detrimental secondary injury cascades can be ascertained. Importantly, knowledge acquired from monitoring and investigation of these injury processes is expected to facilitate the future development of novel treatment strategies. Recently, improved intracranial monitoring was stressed as one of the key areas of research by a commissioned article devoted to TBI in a Lancet Neurology editorial (8).

In this Research Topic, a number of authors from several key centers of excellence worldwide have shared their knowledge on the monitoring of acute brain injuries. These contributions provide updated knowledge of the pathophysiology of TBI and other acute brain injuries, as well as of refining patient management strategies. The overall goal of improving patient outcomes by the detection of deleterious secondary injury processes occurring in the injured brain.

## Neuroimaging

- Stovell et al. reviewed the monitoring of brain metabolism using magnetic resonance spectroscopy (MRS) in acute TBI. While this technique is still in its infancy, it holds much promise as increasing evidence suggests that deranged metabolism is a major exacerbating factor following brain injury, especially in relation to mitochondrial dysfunction. The MRS technique shows potential for the evaluation of specific brain regions where tissue fate may be analyzed granularly in both acute and chronic phases of brain injury.
- Rostami et al. evaluated the correlation between cerebral blood flow and metabolism in patients following SAH monitored using Xenon-CT in combination with cerebral microdialysis (CMD). They observed that reduced global blood flow the first 3 days post-SAH was significantly associated with brain metabolism monitored using CMD, specifically high levels of lactate indicating anaerobic metabolism. These results suggest that monitoring of cerebral blood flow and brain neurochemistry could aid physicians and guide treatments, such as optimizing cerebral perfusion pressure (CPP) and adjusting oxygen and glucose substrate levels in SAH patients.

## Coagulation

- Lindblad et al. evaluated multiple electrode aggregometry (Multiplate^®^) used to assess platelet functions in NICU-treated TBI patients. Although platelet count may be normal, several studies have shown that they do not function properly following TBI. Thus, the primary hemostasis may be altered, specifically by an impaired response of the arachidonic acid (ASPI) receptor. The authors could show that 65% of the included TBI patients had abnormally low ASPI values. This was associated with a more unfavorable outcome, although interestingly not with hemorrhagic progression. These results highlight the need for future research and validated methods of platelet assessment for patients not on platelet anti-aggregation therapies.

## Clinical Investigations

- Marklund evaluated the current role of the neurological wake-up test (NWT) in neurocritical care monitoring, reviewing the available literature. While intracranial pressure (ICP) and CPP monitoring as well as serum markers indicate that the NWT could induce a mild stress response including increased ICP and the release of stress-related hormones, the consequences of these are not fully understood. While qualitative prospective studies concerning its safety are lacking, the NWT remains an important monitoring tool in selected TBI and SAH patients, preferably in combination with multimodal monitoring.

## Cerebral Microdialysis

- Carteron et al. report on how CMD has allowed them to individualize neurocritical care therapy in TBI and SAH patients. Specifically, CMD may help guide optimization of CPP, blood transfusions, glucose infusions, and brain tissue oxygenation targets. Newer markers include electrolytes, markers of oxidative stress, or endothelial proteins, which may also be measured by CMD in order to better predict outcome or guide treatment.
- Helbok et al. conducted a systematic review on the clinical use of CMD in patients with aneurysmal SAH, focusing on secondary brain injury and clinical outcome. They found that the metabolic changes detected by CMD were associated with early and delayed secondary brain injuries and suggested that CMD be used in conjuncture with other monitoring modalities. In summary, while CMD in SAH is less studied than in TBI, it is an emerging area that might establish itself as a future standard monitor of SAH patients, awaiting additional investigations and multi-center trials.

## Biomarker Monitoring

- Ercole et al. review and summarize several different technologies to monitor molecular signals in TBI. Mass spectroscopy as well as optical spectroscopy also hold a potential to unravel the *in vivo* molecular signatures in TBI patients. Altogether, by introducing an “-omics” approach in TBI, it will be possible to gain further understanding of the systems biology involved.
- Posti et al. reviewed the field of metabolomic monitoring as a diagnostic tool in severe TBI, by investigating different metabolites (fatty acids, amino acids, as well as sugar derivates), and how they are associated with injury severity and clinical outcome. This is a promising field although larger prospective trials are necessary to establish better thresholds and specific metabolic patterns associated with brain injury and patient outcome.
- Thelin et al. analyzed the current literature, focusing on some of the most commonly studied biomarkers including S100B, neuron-specific enolase (NSE), glial fibrillary acidic protein (GFAP), ubiquitin carboxy-terminal hydrolase L1 (UCH-L1), and neurofilament light (NF-L) in order to review serum level dynamics of these proteins following TBI. In aggregate, shorter half-lives were seen for S100B and UCH-L1 in contrast to NSE, GFAP, and especially NF-L that exhibit increased levels for prolonged periods of time post-injury. These differences should be taken into account when assessing the capability of these biomarkers to predict outcome and monitor secondary pathologies in TBI patients.
- Tsitsopoulos et al. reviewed possibilities for short- and long-term monitoring of axonal injury in TBI. Biomarkers, specifically NF-L, is mentioned as a potential diagnostic candidate, as it has been found enriched in myelinated axons and seen to be elevated in serum in patients with radiologically verified DAI. Novel MRI tools are increasingly used for the detection and progress of axonal injury.

## Neuroinflammation

- Zeiler et al. reviewed the literature on CMD and CSF cytokines in both TBI and SAH patients. They could conclude that there is an association with elevated levels of certain cytokines, among others, interleukin-6 and tumor necrosis factor alpha, and long-term functional outcome. In summary, cytokines as mediators of inflammatory activity can be monitored in the extracellular fluid by CMD and in CSF and appear to be associated with secondary injury pathophysiology and clinical outcome in both TBI and SAH.
- Thelin et al. reviewed several modalities that are used to monitor the neuroinflammatory response following acute brain injury, focusing on TBI and SAH. In summary, the authors suggest a multimodal monitoring approach in order to improve the understanding of the neuroinflammatory response following acute brain injury to determine its role for tissue as well as patient outcome.
- Vink et al. summarize the role of Substance P in the secondary pathophysiology of TBI, reviewing its role in the neurogenic inflammation and association to ICP increases as observed in several of their studies. In summary, the role of substance P in neurogenic inflammation following TBI is increasingly understood and opens up potential future therapeutic targets for patients suffering from acute brain injury.

## Treatment Strategies

- Zoerle et al. address in their review how to rethink the concept of neuroprotection. They suggest that different aspects of TBI care need to be refined and related to TBI pathophysiology, thus requiring individualized care in part guided by advanced monitoring approaches. These monitoring tools could prompt the clinicians to earlier detection of signs that may need treatment. By using these improved neurocritical care interventions, more adequate neuroprotection might be achieved.

## Monitoring Strategies

- Nordström et al. have written a summary on aspects of the physiological and biochemical foundations of NCC treatment strategies. Here, they address the basic concepts of NCC with focus on the key aspects that form the base for the Lund concept, including intracapillary hydrostatic pressure modulation, fluid therapies, and the implementation of brain tissue oxygen- and CMD monitoring.
- Grände critically evaluates the Lund concept, 25 years after its introduction. He focuses his review on temperature, ventilation, nutrition, osmotherapy, decompressive craniectomy, and sedation management. Following the introduction of more advanced monitoring techniques such as CMD, brain tissue oxygen monitoring, and other ICP- and CPP-guiding protocols, many current NCC strategies and targets have gradually approached several of those comprising the Lund concept.
- Figaji reviewed how treatment and monitoring of TBI and spinal cord injury differs between adults and children. As especially younger children have different anatomy and physiology than adults, there are several aspects, which need to be considered by the treating physician. One of the main findings of this literature review is the lack of suitable studies focusing primarily on the pediatric CNS following acute brain and spinal injury. This lack of knowledge could lead to adult thresholds and ranges for intracranial monitoring parameters being used in many pediatric situations, which might be inappropriate or even harmful to this patient population.

## Conclusion

In this research topic, many aspects of the current knowledge and possible future direction of multimodal monitoring in TBI patients are reviewed. Commonly, only limited aspects of the underlying pathophysiology in acute brain injury can and are monitored in the clinical setting. Introducing additional monitoring modalities including parameters such as inflammation markers, multiple metabolites, and a more granular evaluation of structural injuries, a more comprehensive understanding of the evolution of TBI pathology and ongoing secondary injury processes may emerge in the near future. This will require continued large research efforts, but is expected to facilitate development of novel therapeutic options, personalized medicine, and treatment strategies for patients suffering from acute brain injury.

## Author Contributions

ET, DN, AH, and NM confirm being the sole contributors of this work and approved it for publication.

## Conflict of Interest Statement

The authors declare that the research was conducted in the absence of any commercial or financial relationships that could be construed as a potential conflict of interest.
